# Surgical outcomes in an improvised underground operating room during missile attacks

**DOI:** 10.1007/s00068-026-03316-z

**Published:** 2026-09-03

**Authors:** Ilan Y. Mitchnik, Ahmad Essa, Ofer Heinig, Jonathan G. Silberzweig, Yuval Ben Sira, Oded Rabau

**Affiliations:** 1 Orthopaedic Surgery Department, Shamir (Assaf Harofeh) Medical Center, Zerifin, 60930 Israel; 2 Gray Faculty of Medical & Health Sciences, Tel Aviv University, Tel Aviv, Israel

**Keywords:** Improvised operating room, Armed conflict, Adaptation, Missile attack, Patient outcomes

## Abstract

**Purpose:**

Armed conflict disrupts healthcare when hospital infrastructure is vulnerable to missile attacks. During a 12-day missile strike period, our hospital established an improvised operating room (OR) in an underground parking lot. This study evaluated perioperative and short-term outcomes among patients treated in the improvised OR compared with those treated in the hospital’s fortified standard OR.

**Methods:**

This retrospective cohort study included patients undergoing general or orthopaedic surgery during the 12-day conflict. Procedures identified via surgical logs were categorized by OR location (improvised or standard). Demographic, perioperative, and post-discharge data were collected from electronic records. Primary outcomes included operative time, estimated blood loss, transfusions, complications, hospital and ICU length of stay, ED visits, readmissions, and 3-month mortality.

**Results:**

Overall, 74 surgeries took place in the improvised OR and 77 in the standard OR. Patients in each group were comparable in age, sex, comorbidities, and anesthesia. Improvised OR surgery time was longer (mean 68.1 vs. 47.7 minutes, *p* < 0.001). There were no significant differences between groups in terms of blood loss, transfusions, postoperative complications, or hospital length of stay. After 3-month follow-up, there were no significant differences in readmissions or mortality rates.

**Conclusions:**

Establishing an improvised underground OR during an armed conflict enabled maintaining surgical capacity without compromising short‑term patient outcomes. Prolonged operative time likely reflected logistical challenges in supply management.

## Background

In an era marked by escalating global unrest and armed conflict, the safety and continuity of healthcare services are increasingly under threat [1]. Traditional hospital infrastructure, often unprepared for the event of missile attacks, civil upheaval, or sudden surges in casualties, requires innovative approaches to ensure that both patients and healthcare providers remain protected [2, 3].

**Fig. 1:**
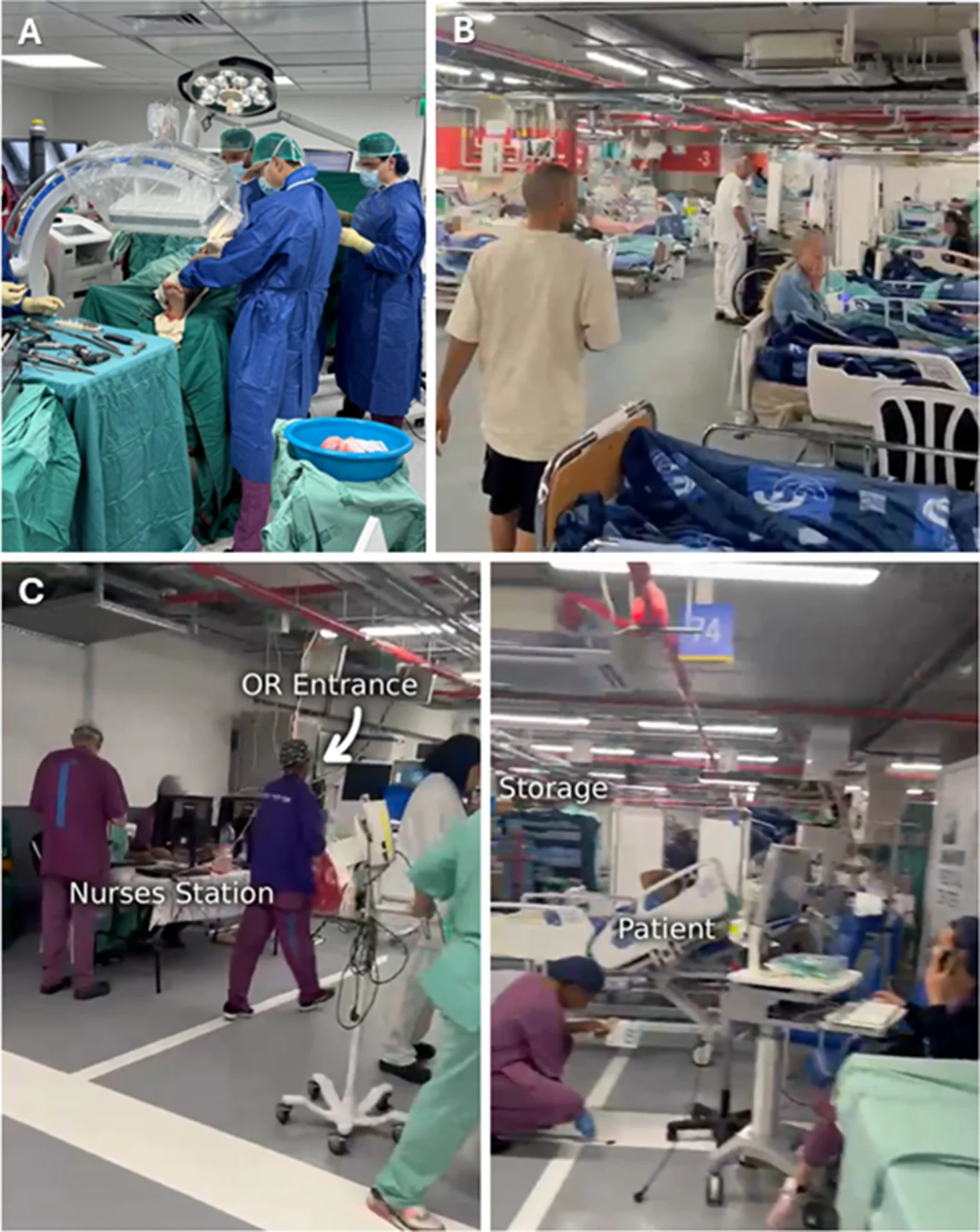
Improvised underground operating room set-up. Improvised operating room set-up in a hospital underground parking lot. **A**: The operating room located four floors below the ground included enough room for an operating bed, ventilating machine, fluoroscopy arm and screen. The room had mobile overhead light sources, oxygen and water sources, and the improvised room closed with sliding doors. **B**: The surgical ward where patients were hospitalized three floors below the ground. **C**: The pre-op holding area located four floors below the ground next to the improvised operating room. Note the limited improvised storage room located between this area and the operating room entrance

Although principles for safe surgery in resource-constrained settings have been described, limited available literature addresses patient outcomes [4]. When standard infrastructure is threatened, hospitals may need to decide, under time pressure, whether selected operations can be safely shifted away from conventional operating rooms (ORs). In June 2025, an escalation of violence erupted as a 12-day conflict of long-range missile attacks began targeting the region, placing pressure on the civilian population. The barrage of missiles challenged the provision of essential healthcare. Hospitals, long aware of their vulnerability in times of conflict, had already taken steps in prior years to reinforce some operating rooms as shelters [2]. Yet, the unprecedented intensity of this conflict compelled many facilities to reduce elective surgeries, prioritizing only urgent and life-saving procedures to prevent overwhelming the few fortified operating spaces available. At our Level 1 trauma center, only one of ten ORs was fully secured against missile strikes. Recognizing the potential for overflow, our hospital leadership adopted an innovative approach, establishing a fully functional improvised OR deep in the underground parking lot, four floors beneath the surface, beyond the reach of missile attacks (Fig. 1). This improvised OR was equipped to match the basic standards of a conventional theater. It included an adjustable operating bed, surgical lighting, a continuous supply of oxygen and water, a modern ventilator for anesthetic management, and sinks for surgical hand washing. The room also opened and closed with automatic sliding doors which were quickly installed. Built rapidly, it enabled uninterrupted surgical care over the entire 12-day conflict.

Once the conflict subsided, our attention turned to the safety and effectiveness of this improvised solution. We undertook this study to evaluate whether patient outcomes in the underground improvised OR compared to those in the fortified standard OR, thereby assessing if our rapid adaptation had truly protected and served our patients in their greatest moment of need.

## Materials and methods

### Setting & participants

The was a retrospective cohort study conducted at a Level 1 trauma center located in a region exposed to continuous missile attacks during a 12-day armed conflict. All consecutive patients undergoing surgical procedures performed by the general surgery or orthopaedic surgery departments during the 12-day conflict period were eligible for inclusion. Elective surgical activity was substantially curtailed during this period; the study population consisted of emergency, urgent, or time-sensitive procedures that the treating teams judged should not be deferred until after the conflict. Patients were identified through institutional surgical logs and cross-referenced with OR records, which indicated whether procedures were performed in the improvised or standard OR. Exclusion criteria included age under one year and procedures that were performed exclusively in one type of OR. For example, spinal surgeries were excluded due to their reliance on intraoperative navigation systems available only in the standard OR. The study was approved by the institutional review board (approval no. ASF-0214-25). This study is reported in accordance with the Strengthening the Reporting of Observational Studies in Epidemiology (STROBE) statement (see supplemental file for checklist).

### Operating room allocation

Allocation to the improvised OR or fortified standard OR followed a pragmatic, realtime decision process. No formal triage protocol was implemented due to the rapid onset of hostilities. During the conflict, allocation decisions were made by the responsible clinical and OR leadership, including the treating surgical team, anesthesia team, and operating-room management, according to clinical urgency, OR availability, anesthetic requirements, required equipment and infrastructure, staffing, and whether the planned procedure could be performed safely in the improvised environment. Procedures requiring equipment, imaging, navigation, or infrastructure unavailable in the improvised OR were preferentially directed to the fortified standard OR. When such procedures had no counterpart in the improvised OR during the conflict, they were excluded from the comparative analysis. Therefore, the study compares outcomes only among the subset of operations for which treatment in either OR setting was considered feasible.

### Variables & measures

Data were extracted from the institutional electronic medical record and included demographic, perioperative, and post-discharge variables. Demographic variables included patient age, sex, Charlson Comorbidity Index (CCI), and American Society of Anesthesiologists Physical Status Classification (ASA) scores. Additional covariates included emergency status (emergency or urgent), procedure type (general surgery or orthopaedic surgery) and anesthesia type (general, spinal, or regional/local). We have also collected data for the lead surgeon experience (attending or resident).

The primary outcomes involved perioperative variables including operative time (minutes), estimated blood loss (milliliters), and the receipt of blood transfusions. Postoperative complications were defined as any documented medical or surgical adverse event during the index admission. When documented, the review included surgical-site infection, wound complications, return to the operating room, and other postoperative adverse events. Hospitalization outcomes included hospital length of stay (LOS), intensive care unit (ICU) LOS, emergency department (ED) visits following discharge, hospital readmissions, and all-cause mortality. Follow-up data were collected through review of all institutional encounters for a duration of three months from the index operation. Procedures were categorized into OR location (standard OR or improvised OR). All variables were compared between these two groups.

### Statistical analysis

The study size was determined by the fixed 12-day conflict period and by the number of consecutive eligible procedures performed during that time; no a priori power calculation was performed. Baseline demographic and clinical characteristics were first compared between the two groups (improvised OR and standard OR). Next, comparison of perioperative and hospitalization outcomes between the two groups were performed. Continuous variables were analyzed using Student’s t-tests or Mann–Whitney U tests, based on distribution. Categorical variables were compared using χ^2^ tests. Standardized mean differences (SMD) were calculated and absolute SMD below 0.10 was considered to indicate negligible imbalance, values between 0.10 and 0.20 suggested a small imbalance, and values greater than 0.20 indicated a potentially meaningful difference. Adjusted comparisons were performed using regression models. Linear regression was used for continuous outcomes and logistic regression for binary outcomes, with effect sizes reported as mean differences (MDs) or odds ratios (ORs), respectively, with 95% confidence intervals (CIs). The primary adjusted models included age, sex, CCI, and anesthesia type. These variables were selected a priori because of their clinical relevance as potential confounders for perioperative outcomes. Additional potentially relevant confounders, including ASA classification, emergency status, procedure type, and surgeon experience, were not included in the primary adjusted models because they would have increased the risk of model overfitting and unstable estimates. Residual confounding from these factors was addressed in a univariate comparison between the two groups. Missing data were not imputed; analyses were performed using available data for each outcome. Statistical significance was defined as a two-sided *p* < 0.05. All statistical analyses were performed using R (version 4.5.2; R Foundation for Statistical Computing, Vienna, Austria).

## Results

**Fig. 2:**
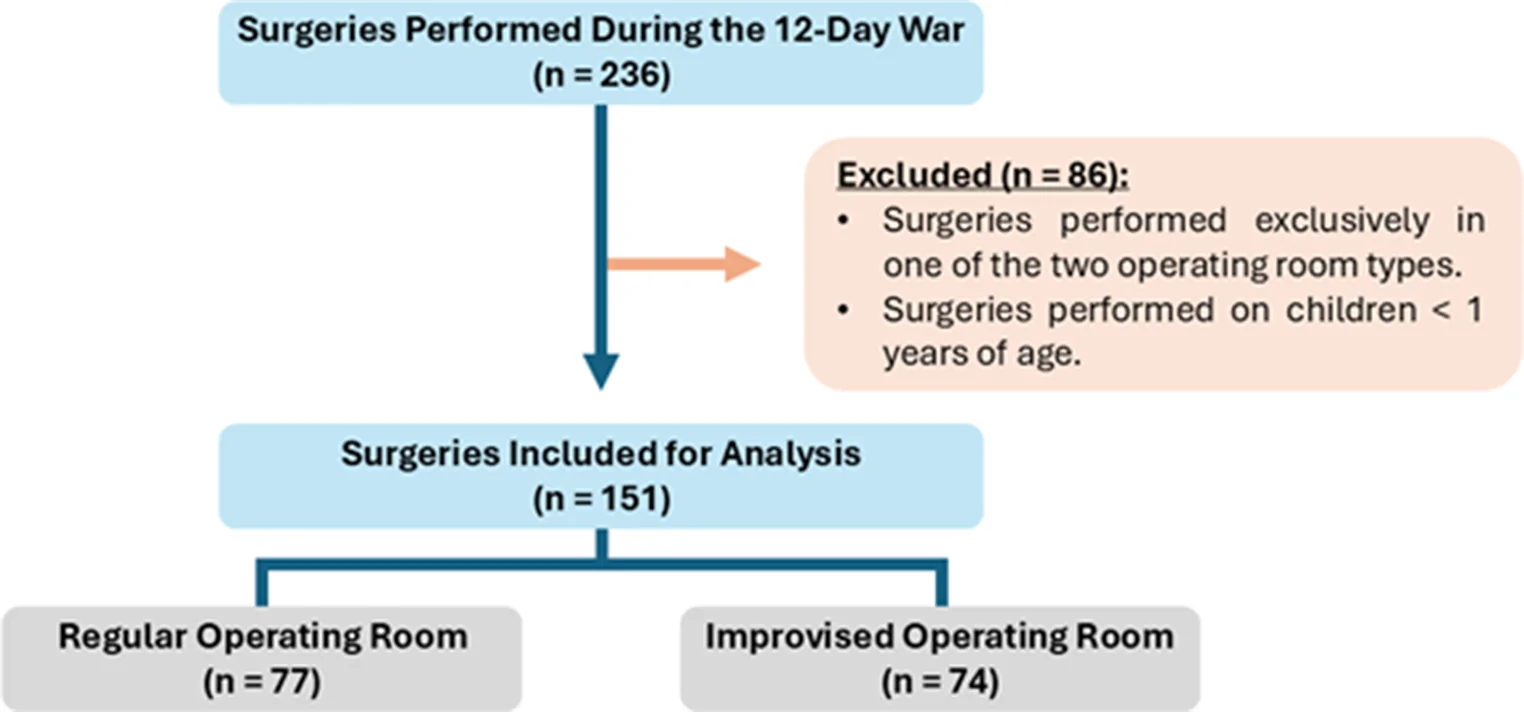
Flowchart of the study’s cohort creation

A total of 236 surgical procedures were performed during the 12-day conflict period. Of these, 85 procedures were excluded because they were surgeries performed exclusively in the standard OR and therefore lacked a comparable counterpart in the improvised OR. The final cohort comprised 151 surgeries, of which 77 (51%) were performed in the standard OR and 74 (49%) in the improvised OR (Fig. 2).

The overall cohort had a mean age of 58.9 years (SD 23.1), and 48.3% were female. Orthopaedic procedures accounted for 61.6% (93/151) of cases, and general surgery for 38.4% (58/151). The two groups were comparable in age, sex, and comorbidities. Anesthesia types were distributed similarly between groups. Table 1 presents the baseline demographic and clinical characteristics of patients according to OR setting.

**Table 1:** Baseline demographic and clinical characteristics

		Standard OR (N = 77)		Improvised OR (N = 74)		Total (N = 151)	SMD	p-value
**Age** (years)							0.22	0.18
Mean (SD)		56.4 (23.6)		61.4 (22.4)		58.9 (23.1)		
Median [Min, Max]		60.0 [3.00, 92.0]		66.0 [3.00, 96.0]		64.0 [3.00, 96.0]		
**Sex** n(%)							0.12	0.57
Females		35 (45.5%)		38 (51.4%)		73 (48.3%)		
**CCI** n(%)							0.06	0.92
$\ge \, 3$		42 (54.5%)		42 (56.8%)		84 (55.6%)		
1–2		13 (16.9%)		13 (17.6%)		26 (17.2%)		
0		22 (28.6%)		19 (25.7%)		41 (27.2%)		
**ASA** n(%)							0.60	0.25
I		16 (20.8%)		13 (17.6%)		29 (19.2%)		
II		27 (35.1%)		31 (41.9%)		58 (38.4%)		
III		22 (28.6%)		26 (35.2%)		48 (31.8%)		
IV		3 (3.9%)		2 (2.8%)		5 (3.3%)		
**Procedure Type** n(%)							0.13	0.52
General Surgery		32 (41.6%)		26 (35.1%)		58 (38.4%)		
Orthopedic		45 (58.4%)		48 (64.9%)		93 (61.6%)		
**Surgeon Experience** n(%)							0.14	0.52
Attending		57 (74.0%)		59 (79.7%)		116 (76.8%)		
Resident		20 (26.0%)		15 (20.3%)		35 (23.2%)		
**Surgery Type** n(%)							0.06	0.85
Emergency		49 (63.6%)		45 (60.8%)		94 (62.3%)		
Urgency		28 (36.4%)		29 (39.2%)		57 (37.7%)		
**Anesthesia Type** n(%)							0.38	0.07
General		61 (79.2%)		51 (68.9%)		112 (74.2%)		
Spinal		5 (6.5%)		14 (18.9%)		19 (12.6%)		
Local\Regional		11 (14.3%)		9 (12.2%)		20 (13.2%)		

OR = operating room; CCI = Charlson Comorbidity Index score; ASA = American Society of Anesthesiologists Physical Status Classification; SMD = Standardized mean difference

Perioperative and hospitalization outcomes are presented in Table 2. Procedures performed in the improvised OR had significantly longer operative durations compared to those in the standard OR (mean 68.1 minutes vs. 47.7 minutes, *p* < 0.001). There were no significant differences between groups in estimated blood loss, perioperative transfusion rates, or the incidence of postoperative complications during the index hospitalization. Hospital and ICU length of stay were similar between groups. Over the three-month follow-up period, no significant differences were observed in rates of return to the emergency department, hospital readmissions, or mortality.

**Table 2:** Comparison of perioperative and hospitalization outcomes

	Standard OR (N = 77)	Improvised OR (N = 74)	p-value
**Peri-Operative Outcomes**			
**Surgery Time** (minutes)			
Mean (SD)	47.7 (32.9)	68.1 (48.5)	**<0.001**
Median [Min, Max]	40.0 [4.00, 193]	59.0 [7.00, 280]	
**Blood Loss** (ml)			
Mean (SD)	41.2 (92.9)	22.2 (49.4)	0.12
Missing	0 (0%)	1 (1.4%)	
**Blood Transfusion** n(%)			
$\ge \, 3$	0 (0%)	1 (1.4%)	0.90
1–2	9 (11.7%)	9 (12.2%)	
0	67 (87.0%)	64 (86.5%)	
Missing	1 (1.3%)	0 (0%)	
**PostOp Complications** n(%)			
Yes	8 (10.4%)	9 (12.2%)	0.93
**Hospitalization Outcomes**			
**Hospital LOS** (Days)			
Mean (SD)	7.19 (12.5)	7.68 (16.1)	0.84
**ICU LOS** (Days)			
Mean (SD)	0.273 (1.90)	0.216 (1.05)	0.82
**Return to ED** n(%)			
Yes	12 (15.6%)	14 (18.9%)	0.74
**Readmissions** n(%)			
Yes	5 (6.5%)	7 (9.5%)	0.71
**Mortality** n(%)			
Yes	3 (3.9%)	5 (6.8%)	0.49

OR = operating room; PostOp = post-operative; LOS = length of stay; ICU = intensive care unit; ED = emergency department

**Fig. 3:**
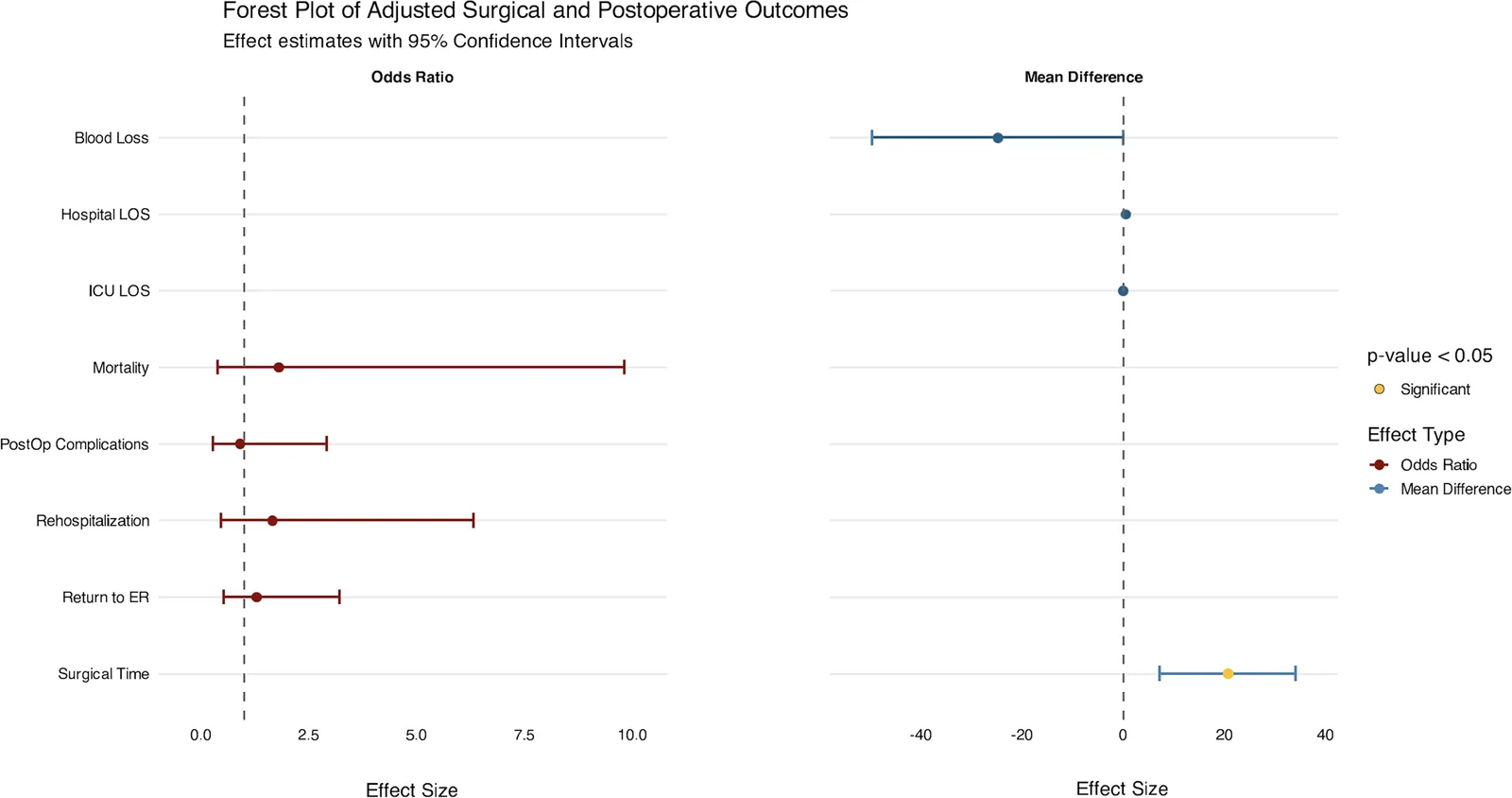
Forest plot of adjusted surgical and postoperative outcomes comparing regular and improvised operating rooms during the 12-Day war

Adjusted comparisons between the improvised and standard OR groups are presented in Table 3. After adjustment for age, sex, CCI, and anesthesia type, procedures performed in the improvised OR remained significantly longer, with a mean difference of 20.7 minutes (95% CI: 7.28 to 34.11, *p* = 0.002). No other adjusted comparisons reached statistical significance. Figure 3 presents a forest plot visualizing the adjusted outcome analysis across all endpoints.

**Table 3:** Adjusted comparison of surgical and postoperative outcomes between patients operated in standard vs. Improvised operating rooms

Continuous Outcomes	Mean Difference	95% Confidence Interval	p-value
Surgery Time	20.70	7.28 to 34.11	0.002
Blood Loss	−24.78	−49.64 to 0.06	0.051
Hospital LOS	0.52	−4.16 to 5.21	0.83
ICU LOS	−0.05	−0.57 to 0.46	0.83
**Categorical Outcomes**	**Odds Ratio**	**95% Confidence Interval**	**p-value**
PostOp Complications	0.91	0.28 to 2.92	0.88
Return to ED	1.29	0.53 to 3.22	0.57
Readmissions	1.66	0.47 to 6.33	0.44
Mortality	1.81	0.39 to 9.82	0.45

OR = operating room; PostOp = post-operative; LOS = length of stay; ICU = intensive care unit; ED = emergency department

## Discussion

The most important finding of this study is that setting up an improvised OR in a hospital underground parking lot was not associated with an increased risk of perioperative or hospitalization complications. The only difference found was a significantly increased operating time in the improvised OR. These findings suggest that, within a tertiary hospital and among procedures considered feasible in either environment, an improvised underground OR may help preserve surgical capacity during armed conflict.

Few studies exist for comparison, but it’s established that less-equipped ORs, such as those in Africa, face infection rates up to 50% [5]. Operating outside standard ORs raises concerns about sterility, especially since even traditional ORs can lose sterility with frequent door openings [6–8]. The World Health Organization guidelines for safe OR set-ups include having trained personnel, clean water, consistent light source, consistent suction, supplemental oxygen, functioning surgical equipment and sterile instruments [9]. These core requirements form the foundation for maintaining patient safety, even when surgeries must take place outside of traditional operating theaters [10–12]. Supporting this principle, a study from Ghana demonstrated that converting a storage room into an operating space for hand surgeries under local anesthesia successfully increased surgical capacity [13]. Importantly, the authors reported that infection rates did not rise when basic sterile protocols were followed. This remained true even though the converted space lacked typical OR features such as positive pressure and built-in utilities. Similarly, in this study we did not observe an increase in perioperative complication rates, likely since core sterility protocols were maintained.

The longer surgery times may have resulted from logistical challenges in transporting supplies. This explanation is hypothetical and relies on the following observation: Over the 12-day conflict period, it was clear that booking the operating room required specifying every instrument and supply so technicians could retrieve them from storage. If additional equipment was required during a procedure, a nurse needed to walk from the parking lot to the surgical storage to collect it. Furthermore, the use of a temporary supply storage area in the parking lot complicated matters, as nurses were less familiar with item locations, which likely contributed to further delays. Although previous studies have consistently demonstrated that prolonged surgeries are associated with higher complication rates [14, 15], this study did not identify an increase in complications despite extended operative times.

Our study suggests that creating an improvised OR in an underground parking lot is both feasible and safe for patients. We advise other hospitals to focus on thorough logistical preparation, especially regarding medical supplies, as this may help reduce operative times. Our findings are particularly relevant to conflict zones worldwide that face increasing threats from missile attacks on civilian areas. As far as we know, this is the first report documenting the use of unused hospital spaces for major surgeries during missile attacks. However, the improvised OR was located inside a tertiary hospital with modern infrastructure, trained staff, anesthesia support, sterile processing, blood-bank access, imaging, ICU capacity, and backup fortified OR availability. These results should not be generalized directly to austere field hospitals, facilities without reliable utilities, or settings lacking hospital-level support.

The primary limitation of our study lies in its retrospective nature, which may result in incomplete documentation within patient records. Secondly, complex cases requiring infrastructure available only in the standard OR were excluded from the analysis. This exclusion limits the generalizability of our findings, as the results may not fully represent the spectrum of emergency surgeries performed under wartime conditions. Third, we are unable to establish that increased operative time was attributable to logistical factors, as these were not assessed as confounders. Furthermore, the scope of this analysis is restricted to a three-month follow-up period, leaving long-term complications yet to be determined. Finally, the cohort was relatively small, adverse events were uncommon, and several confidence intervals were wide. Thus, a clinically important difference could therefore have been missed because of type II error.

In conclusion, establishing an improvised underground OR during an armed conflict allowed the hospital to maintain surgical capacity without compromising short‑term patient outcomes. Prolonged operative time likely reflected logistical challenges in supply management. These findings demonstrate that essential surgical capacity can be maintained in improvised hospital environments when standard ORs are inaccessible during emergencies.

## Data Availability

The data that support the findings of this study are available from the corresponding author upon reasonable request.
